# Enhanced 3D imaging accuracy using curved sensors: a simulation-based approach

**DOI:** 10.1038/s41598-026-48047-8

**Published:** 2026-04-21

**Authors:** Sayyed Mohammad Emam, Hamidreza Daliri, Abolfazl Foorginejad, Siamak Khatibi

**Affiliations:** 1 Department of Mechanical Engineering, Faculty of Engineering, Ardakan University, P.O. Box 184, Ardakan, Iran; 2 Department of Mechanical Engineering, Birjand University of Technology, Birjand, Iran; 3 Department of Technology and Aesthetics, Blekinge Institute of Technology, 371 79 Karlskrona, Sweden

**Keywords:** 3D imaging, Curved sensor, Optical distortion, Image quality, Measurement accuracy, Engineering, Optics and photonics

## Abstract

Three-dimensional (3D) modeling is a key requirement in computer vision, yet achieving high-speed and high-quality image acquisition remains challenging. Flat image sensors, commonly used in 3D scanners, suffer from edge blurring due to misalignment between the sensor plane and the curved focal surface of the lens. While complex lens designs can mitigate this issue, they increase manufacturing costs, weight, and optical distortions. This study investigates the advantages of curved sensors in improving imaging performance. Through software-based modeling and simulation, the study compares curved and flat sensors by analyzing key optical errors, including astigmatism, distortion, spherical aberration, and coma. The results indicate that curved sensors significantly enhance image quality and measurement accuracy. Notably, measurement errors are reduced by 44.68% compared to flat sensors. The findings highlight curved sensors as a promising alternative for high-precision 3D imaging, offering improved edge detection and dimensional measurement accuracy while reducing optical distortions.

## Introduction

A three-dimensional (3D) scanner is a device that analyzes real-world objects or environments and collects digital data based on their probable shape and appearance. The primary function of a 3D scanner is to generate a point cloud of an object’s surface and create a virtual model. In modern optical design, minimizing optical aberrations is key to achieving high performance. Optical aberrations are classified into two main categories: monochromatic and chromatic. Monochromatic aberrations include spherical aberration, coma, astigmatism, field curvature, and distortion, while chromatic aberrations consist of longitudinal and lateral components. Reducing these seven types of aberrations remains one of the major challenges in optical engineering.

A thorough assessment of the performance and limitations of 3D scanners is essential for selecting the optimal system for a given application. Errors in 3D scanning devices arise from both hardware and software limitations. One of the most critical hardware components in 3D scanners is the camera, which directly influences dimensional measurement accuracy. The camera sensor, a key element, consists of an array of pixels that capture light reflected from objects after passing through the lens. This light is recorded on a flat sensor plane positioned at the focal length of the lens. Similar to the human eye, the camera lens focuses an inverted image onto the sensor. However, unlike the human eye, where the light-sensitive surface (the retina) is curved, conventional flat sensors introduce measurement errors, particularly at the edges of the image. This results in reduced image quality outside the optical axis, as the focal depth of a lens cannot accommodate the differential distance between the sensor plane and the curved focal surface.

To mitigate this issue, complex lenses have been proposed to optimize image projection onto flat sensors. However, such approaches increase manufacturing costs, add weight to the lens system, and introduce additional optical distortions.

This study aims to investigate the potential of curved sensors by modeling and simulating a sensor shape that aligns with the actual focal surface of a simple lens. The primary objectives are to enhance image quality and sharpness, expand the camera’s field of view, and improve edge detection and dimensional measurement accuracy using conventional, low-cost equipment. The proposed scanner system is first analyzed theoretically, and mathematical relationships for evaluating the accuracy improvement in the designed hardware are formulated. Subsequently, a software-based simulation of the curved sensor is conducted, and the theoretical results are validated.

## Related work

The physical and geometric characteristics of the scanned object influence measurement accuracy and impose limitations on the scanner performance^1,2^. Hardware errors affecting accuracy include lens precision, errors in converting analog images to digital format, and errors in data storage^3^. Another key hardware limitation affecting scanner accuracy is the resolution or spatial resolution of the camera. Since digital cameras store image planes as an array of pixels, each point position in the image is estimated based on the center of the pixel where it is located. This error, known as quantization error, persists in later processes such as modeling and depth reconstruction^4,5^. The larger the pixel area, the greater the positional estimation error of scene points. Therefore, finding the optimal pixel size, which can be determined through camera simulation, is essential. However, since pixel size depends on the sensor manufacturing technology, it cannot be considered a direct method for improving depth reconstruction accuracy^6^. One of the critical challenges in image sensor design is enhancing the fill factor, which represents the ratio of the light-sensitive area to the total pixel dimensions. In evaluating smart image sensor performance, reducing power consumption and increasing the effective fill factor of each cell are crucial aspects. Novel structural designs in image sensor cells have significantly improved the effective area of each pixel^7^.

In recent years, digital cameras have seen remarkable advancements in resolution and low-light performance. However, in terms of precision and structure, they still lag behind the visual system of living organisms. Curved image sensors, inspired by the eyes of animals and insects, represent an innovative breakthrough in the next generation of digital cameras. Fisheye cameras, due to their spherical lens geometry and hemispherical retina, provide a wide field of view. However, manufacturing curved sensors remains a challenging process due to the complexity of production^8,9^. The use of curved sensors for astronomical applications, which better align with curved focal planes, has revolutionized optical systems, significantly enhancing their scientific potential^10^. Transforming the sensor surface from flat to curved increases the amount of light received across different wavelengths and reduces the number of optical components required to correct aberrations. This advancement enables high-resolution tracking of celestial bodies within the solar system and precise mapping of distant galaxies. Additionally, stereo cameras equipped with curved pixel arrays require simpler optical lenses and mechanical components than flat sensors, improving imaging speed and reducing overall camera weight^11^.

Software-based studies have demonstrated that sensors with hexagonal pixel arrays outperform those with square arrays in edge detection^12^. Hexagonal arrays increase data density per pixel unit and reduce computational complexity in three-dimensional measurement calculations^13^. This symmetrical configuration enhances computational efficiency in motion detection, edge recognition, and orientation estimation, which often require comparing intensity differences between neighboring pixel cells.

The Petzval curve approximates the focal surface of light reflected from a camera lens. Designing advanced lenses to project images onto flat sensors presents a fundamental challenge, often leading to astigmatism due to the curvature of the Petzval field^14^. If curved sensors could be manufactured following the Petzval curve, they could significantly improve optical systems, including three-dimensional scanners, by compensating for field curvature errors and reducing astigmatism. Additionally, the required lens design for a curved sensor would be more symmetrical and geometrically simpler, making the manufacturing process more feasible^15,16^.

Despite advancements in image sensor performance, relatively little research has focused on overcoming the limitations of flat sensor arrays. Studies suggest that curved sensors can be fabricated using integrated silicon structures^17^. This approach could lead to the development of compact cameras with enhanced optical performance. One proposed design features a hexagonal pixel array mounted on a parabolic curved surface, improving image quality^18^. Imaging with this parabolic sensor, compared to flat sensors, demonstrates better light uniformity and an increased field of view.

While prior studies, such as Xu et al.^19^., have demonstrated the optical advantages of curved sensors in reducing aberrations through simulation of complex lens systems, they primarily focus on qualitative improvements in image quality without addressing the practical challenges of sensor calibration in metrology applications. For instance^19^, reports significant reductions in astigmatism (from 0.04 mm to 0.02 mm) and distortion (from 3 to 0.5%), but does not propose a method to integrate curved sensors into existing calibration pipelines, limiting their applicability in precision 3D measurement tasks. This study fills this gap by introducing a novel calibration approach that adapts traditional flat-sensor techniques to curved geometries via spherical coordinate transformations, enabling accurate dimensional metrology in simulation environments. Unlike^19^, which relies on predefined lens prescriptions without exploring calibration errors, our work quantified measurement accuracy improvements and validated them against real-world-like distortions.

In this study, through software simulation and the transformation of the camera sensor from flat to curved, improvements in edge detection accuracy and dimensional extraction from scans were examined. By replacing the conventional flat sensor with a curved one, the increased sensor field of view minimizes the effects of out-of-range reflected rays, which previously reduced image clarity at the edges and compromised dimensional accuracy.

## Optical aberrations

Optical aberrations in the path of light after refraction through a lens or reflection from a mirror degrade the quality of the final image in optical instruments and systems. To enhance image quality or dimensional measurement accuracy, these aberrations must be minimized or corrected. This paper discusses various optical aberrations, with a particular focus on field curvature, which is central to the scope of this study.

The systematic study of optical aberrations was pioneered by Philipp Ludwig von Seidel, and these aberrations are sometimes referred to as Seidel aberrations. Optical aberrations are broadly classified into two categories: monochromatic and chromatic^20^. Monochromatic aberrations include spherical aberration, coma, astigmatism, field curvature, and distortion, while chromatic aberrations encompass longitudinal and lateral chromatic aberrations. Minimizing all seven aberrations can be achieved by combining multiple lenses; however, this approach increases the size, cost, and weight of the imaging system. Consequently, a key challenge for researchers is to simplify lens systems without compromising image clarity.

### Field curvature aberration and the Petzval curve

**Fig. 1:**
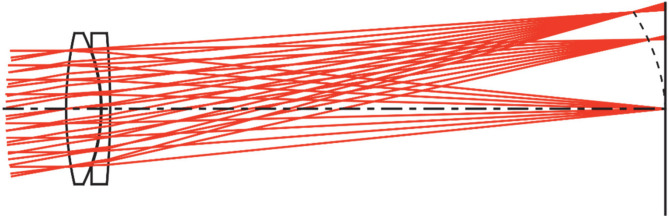
Convergence behavior of ray fans at different object angles focusing on the Petzval surface (dashed line) before reaching the image plane (solid line)^21^.

Field curvature, also known as Petzval field curvature, is a monochromatic aberration that causes the image plane to form a curved surface rather than a flat plane, resulting in focus variations across the field of view. This aberration, derived mathematically by Joseph Petzval, arises when a lens focuses light rays onto a curved surface, known as the Petzval surface, instead of a flat plane. Consequently, achieving uniform focus across the entire image plane becomes challenging. As shown in Fig. 1, field curvature causes the image to form on a curved (Petzval) surface.

**Fig. 2:**
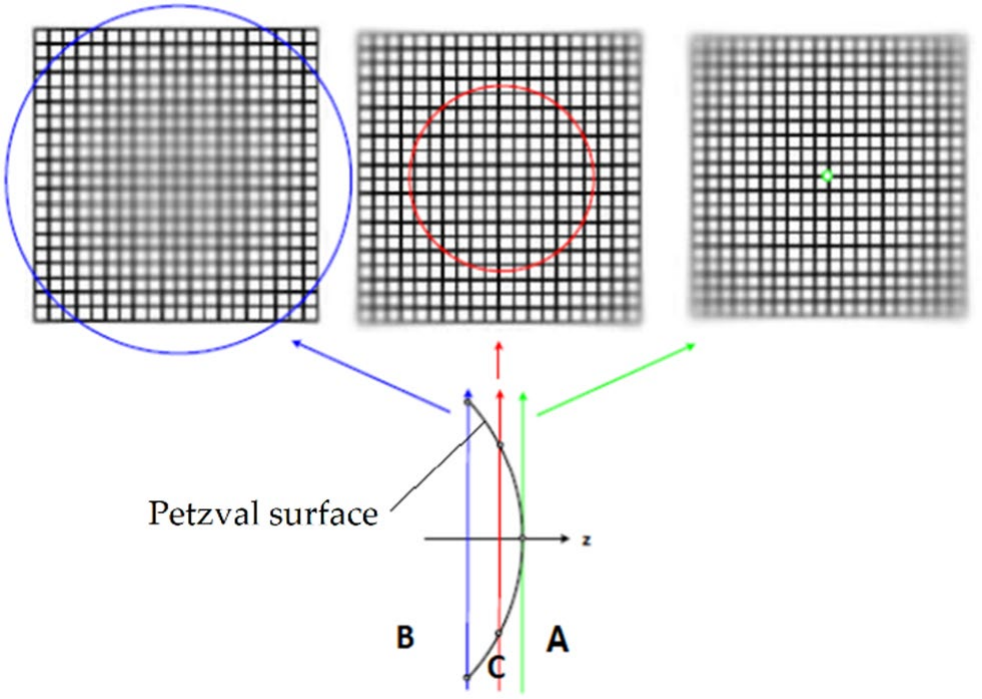
Performance of the flat sensor in various positions relative to the Petzval surface.

In most cases, the results in a forward-curved image surface where only the central region appears sharp, while the peripheral areas are blurred. This issue stems from the inherent curvature of optical elements. Since digital camera sensors are flat, they cannot capture the entire image in full focus. As shown in Fig. 2, positioning the image plane at location A produces a sharp image only at the center, with blurred peripheral regions. At location B, only the peripheral regions are sharp, and at location C, only the intermediate field regions are in focus. To achieve a sharp image across the entire field of view, the image plane would need to conform to a curved surface matching the field curvature or Petzval surface. The most effective method to eliminate field curvature aberration involves using corrective lenses that project the image onto a flat sensor. Alternatively, employing a curved sensor that matches the curvature of the Petzval surface can address this issue.

## Simulation and performance analysis

In this study, using the optical design software Code V^22^, two optical systems—one equipped with a flat sensor and the other with a curved sensor based on the radius of curvature of the Petzval surface—were separately evaluated and analyzed, and their results were compared.

### Specifications and layouts of a triplet lens system

For the simulation, the parameters shown in Table 1, extracted from the example presented in study^23^, were used. The lens system under consideration consists of three lenses, with the geometric and physical specifications of this system for curved sensor provided in Table 2. It should be noted that the lens prescription data for the flat sensor were selected similar to Table 2, with the difference that the radius of the seventh surface was chosen to be infinity.

**Table 1:** Specifications related to the camera’s intrinsic parameters.

Parameters	Specifications
F-number	2.0
Focal length	17.6 mm
Image height	3.6 mm
Sensor type	1/2.5″
Sensor dimensions	5.76 × 4.28 mm
Resolution	2592 H×1944 V
Pixel size	2.2 μm×2.2 μm

**Table 2:** The lens prescription data for a curved sensor^23^.

Surface	Radius (mm)	Thickness (mm)	nd	vd
1	13	3.57	1.618	63.4
2	− 143.5	3.09		
3	− 23.62	0.75	2.001	29.13
4	60.95	0.8		
Stop	Infinity	3.58		
6	11.5	3.75	1.4971	81.56
7	− 15.88	10.49		

**Fig. 3:**
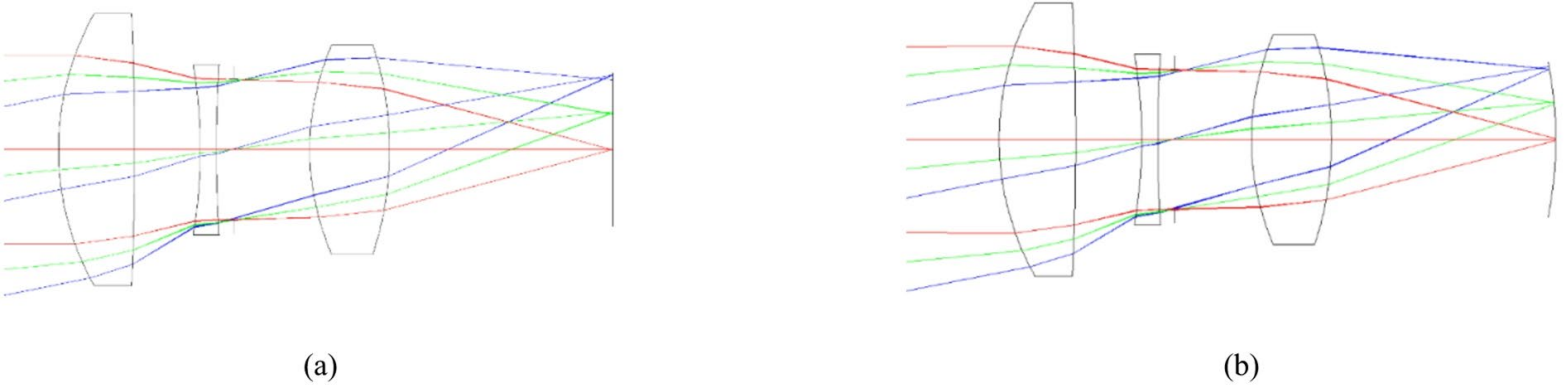
Comparison of triple lens implementations: (**a**) conventional design using a flat sensor, (**b**) a curved sensor with field curvature correction according to Table 2.

The selection of glass material is based on two key parameters, nd and vd, which represent the refractive index and dispersion, respectively. The variation of a material’s refractive index with wavelength is known as its dispersion. The degree of chromatic aberration of a lens is indicated by the Abbe number or *V*-number^21^. It should be noted that the stop surface in Table 2 represents the position of the diaphragm relative to the other lenses in the optical system. Figure 3 illustrates the lens systems designed in this study. This figure demonstrates how light rays, deviated at angles of 0, 7, and 10 degrees relative to the optical axis, affect the lens systems.

**Fig. 4:**
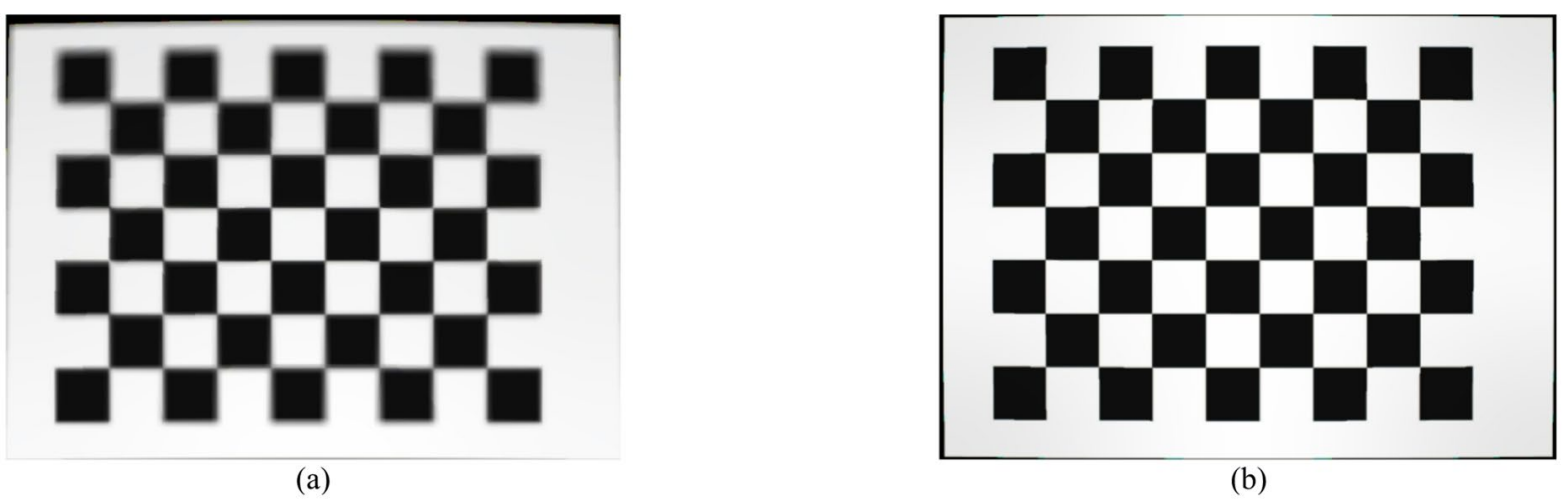
Comparison of images captured from (**a**) flat and (**b**) curved sensors of the same scene under similar conditions.

Figure 4 presents an image of a calibration plane captured under identical conditions using both the flat and curved sensors developed in this study. It can be observed that the image captured by the flat sensor, as shown in Fig. 4a, exhibits high clarity in the central region but becomes blurred at the corners due to field curvature aberration. In contrast, the image obtained from the curved sensor, as seen in Fig. 4b, maintains uniform high clarity across the entire field of view. It is entirely predictable that this difference will negatively impact the measurement accuracy of the flat sensor, leading to increased measurement errors.

### Specifications and layouts of a complex lens system

In conventional cameras, more complex lens systems—typically comprising more than three lenses—are employed to mitigate errors arising from radial distortion and field curvature (Petzval). Consequently, a simulation experiment was designed to compare the measurement accuracy of curved and flat sensors within such a complex lens system. To facilitate software simulation and validate the modeled optical systems, lens specifications as defined in reference^19^ were utilized. Tables 3 and 4 present the design parameters for the curved and flat sensors, respectively, including the radius of curvature of the lens surfaces, the distances between optical components, and the types of glass used for the lenses.

**Table 3:** Prescription data for the optical system with a curved sensor^19^.

Surface	Radius (mm)	Thickness (mm)	Glass
Object	Infinity	Infinity	
1	115.34	12	HFK61_CDGM
2	464.91	10	
3	88.72	10	HFK61_CDGM
4	241.04	10.51	
5	54.25	7.02	HFK61_CDGM
6	152.88	1.89	
7	420.92	5	TF3_CDGM
8	44.37	7.11	
Stop	Infinity	1	
10	191.72	3	TF3_CDGM
11	36.93	5.56	HLAK50_CDGM
12	445.17	24.52	
13	77.58	12	HFK61_CDGM
14	−1099.24	21.79	
15	160.91	13.26	HFK61_CDGM
16	−110.09	15.65	
Image	−84.04	0	

**Table 4:** Prescription data for the optical system with a flat sensor^19^.

Surface	Radius (mm)	Thickness (mm)	Glass
Object	Infinity	Infinity	
1	85.75	8.59	HLAF53_CDGM
2	117.84	10	
3	86.57	9.86	HFK61_CDGM
4	285.69	4.28	
5	64.28	7.43	HFK61_CDGM
6	182.54	2.59	
7	−563.70	5	TF3_CDGM
8	44.1	9.77	
Stop	Infinity	13.2	
10	89.6	3	TF3_CDGM
11	32.72	13.88	HZK3_CDGM
12	136.14	4.34	
13	63.43	14.75	HFK61_CDGM
14	−191.36	3.71	
15	218.4	10.25	HFK61_CDGM
16	−180.11	40.21	
17	−36.40	5.65	HZK9A_CDGM
18	−339.39	3	
Image	Infinity	0	

**Fig. 5:**
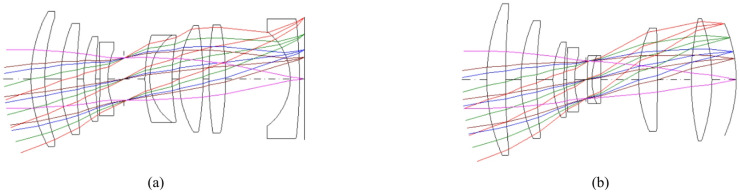
Lens implementations of (**a**) a flat sensor and (**b**) a curved sensor according to Tables 3 and 4.

In Tables 3 and 4, the fourth column specifies the glass type used for the lenses, as provided in the CDGM glass catalog. Figure 5 illustrates the flat and curved lens systems simulated in this study using the CODE V software. As observed, complex lens assemblies are employed to enhance image quality and eliminate aberrations.

It should be noted that the lens prescriptions in Tables 3 and 4 differ because each optical system was independently optimized for its respective sensor geometry (flat or curved), following the design approach presented in the source reference^19^. This is standard practice in optical engineering when comparing complete imaging systems. For the simpler triplet lens (Table 2), however, the lens elements are identical and only the image surface radius is changed from infinity (flat sensor) to the Petzval curvature (curved sensor), allowing a direct evaluation of sensor shape alone.

According to the results presented in reference^19^, the maximum astigmatism deviation is 0.04 mm for the flat sensor and 0.02 mm for the curved sensor. Consequently, the precision of focusing light rays in the horizontal and vertical planes at a single point is twice as high for the curved sensor compared to the flat sensor. The maximum distortion deviation is 3% for the flat sensor and 0.5% for the curved sensor, resulting in a 2.5% improvement in image quality due to reduced distortion and barrel effects in the curved sensor compared to the flat sensor. The reduction in spherical aberration enhances image quality in the curved sensor by approximately four times compared to the flat sensor. The deviation of the farthest points from the optical axis relative to their actual position is 0.02 mm for the flat sensor and 0.005 mm for the curved sensor. Thus, the improvement in image quality due to reduced coma aberration in the curved sensor is approximately four times greater than in the flat sensor.

## Comparison of measurement accuracy between flat and curved sensors

In the previous section, various sources of error that degrade the image quality produced by flat and curved sensors were analyzed and compared. To date, the improvement in measurement accuracy of curved sensors has not been investigated by researchers. Therefore, in this study, a complementary simulation experiment was designed relative to reference^19^, to compare the three-dimensional measurement accuracy of these two sensor types, which is described in detail below. It should be noted that the calibration process for the curved sensor was modeled in a novel and innovative manner in this study, with the relevant mathematical relationships presented.

### Camera calibration

Camera lenses and imaging systems inevitably deviate from the ideal pinhole model due to manufacturing tolerances and assembly misalignments. In practice, real camera optics exhibit lens aberrations and distortions that must be explicitly modeled. These hardware imperfections introduce image distortions—for example, manufacturing irregularities in the sensor or lens assembly can produce perspective distortion that degrades measurement accuracy. Consequently, precise camera calibration becomes essential in any dimensional measurement system. Calibration characterizes the optical path and corrects such distortions, thereby enabling accurate mapping of image measurements to real-world units.

**Fig. 6:**
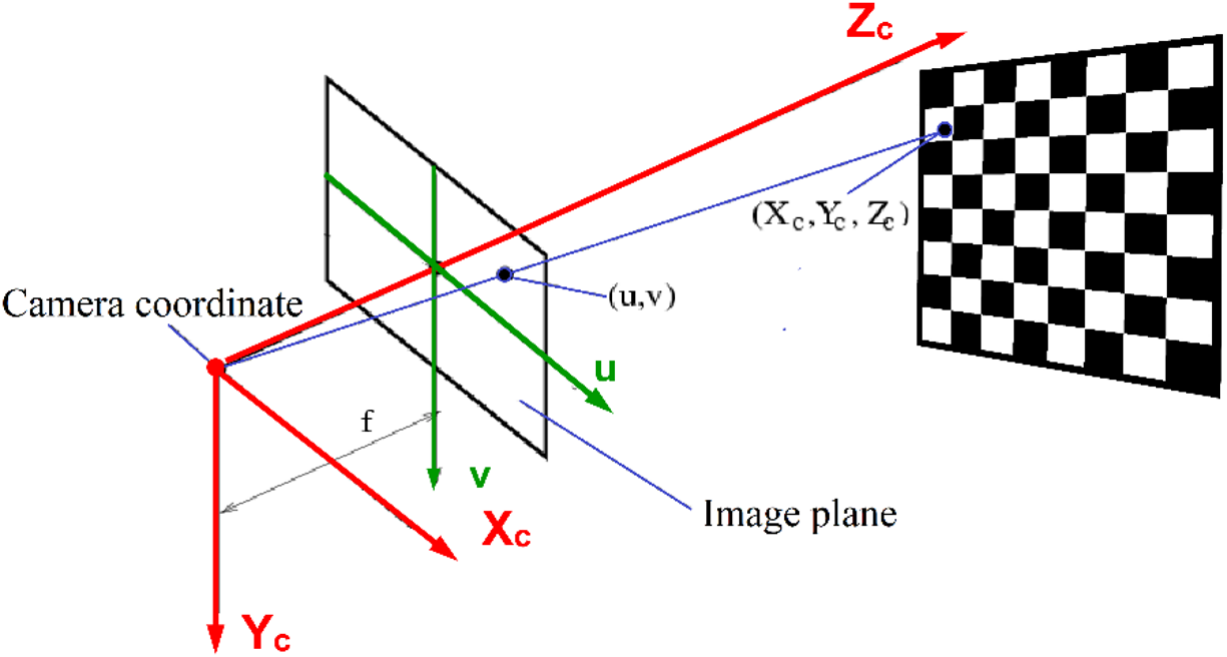
Schematic of perspective projection.

Camera calibration is defined as the process of determining the relationship between three-dimensional world coordinates and their corresponding two-dimensional pixel coordinates in the image. The outcome of calibration includes the camera’s intrinsic parameters (focal length, principal point, lens distortion coefficients, etc.) and extrinsic parameters (the camera’s pose relative to the world frame). To estimate these parameters, one must know correspondences between known 3D points and their 2D image projections. In practice, this is achieved by capturing multiple images of a calibration target with precisely known geometry. As illustrated in Fig. 6, the intrinsic and extrinsic parameters can thus be obtained from several images of a planar calibration pattern (such as a chessboard) viewed from different poses.

Using the principle of similar triangles, we derive the following relation:

1 $$\:u=f\frac{{X}_{C}}{{Z}_{C}},\:v=f\frac{{Y}_{C}}{{Z}_{C}}$$

In the above equation, *u* and *v* represent the image coordinates of a point on the image plane, while (X_C_, Y_C_, Z_C_) denote the coordinates of the same point with respect to the camera’s coordinate system. The focal length is denoted by *f*. Accordingly, the correspondence between the coordinates in the camera coordinate system and those on the image plane can be computed by:

2 $$\:\left[\begin{array}{c}u\\\:v\\\:1\end{array}\right]=\frac{1}{{Z}_{C}}\left[\begin{array}{c}f\\\:0\\\:0\end{array}\:\begin{array}{c}0\\\:f\\\:0\end{array}\:\begin{array}{c}0\\\:0\\\:1\end{array}\:\begin{array}{c}0\\\:0\\\:0\end{array}\right]\left[\begin{array}{c}\begin{array}{c}{X}_{C}\\\:{Y}_{C}\\\:{Z}_{C}\end{array}\\\:1\end{array}\right]$$

**Fig. 7:**
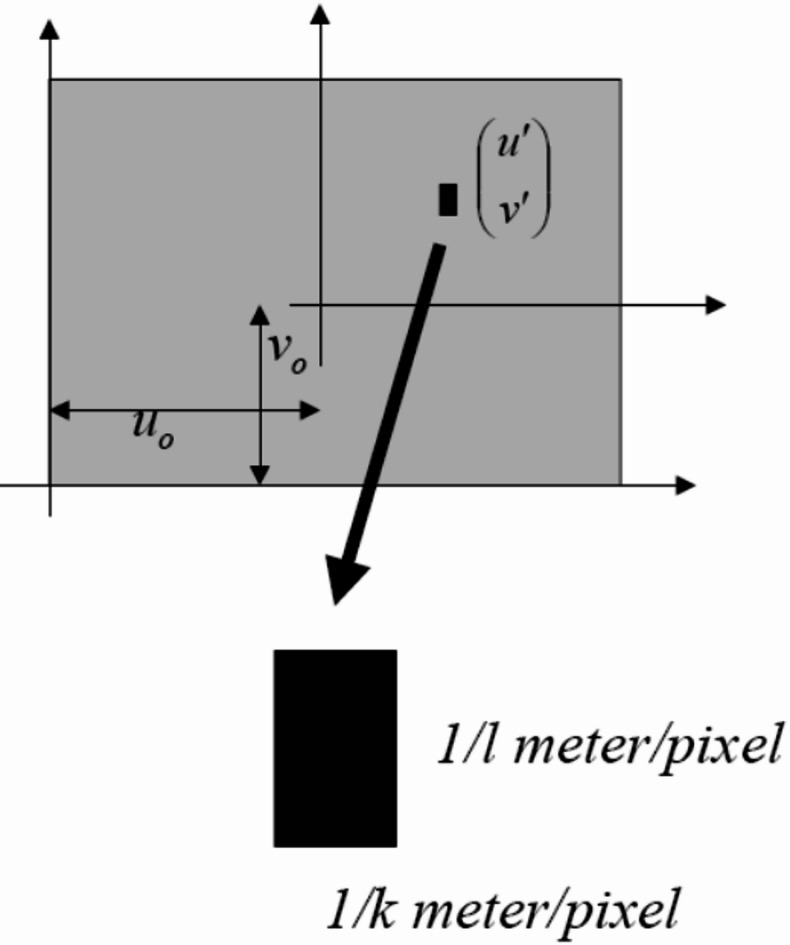
Pixel elongation and deviation from square shape.

Due to factors such as the uncertainty in determining the exact location of the image center relative to the optical axis, the rectangular or square shape of pixels, and the non-orthogonality of sensor axes or edges, additional parameters must be considered in Eq. (2). For instance, as illustrated in Fig. 7, if the pixel edges are transformed into a rectangular shape with scaling factors *k* and *l.*

The updated positions of the points, taking into account the location of the image center (*u*_0_, *v*_0_) can be calculated by:

3 $$\:u=\alpha\:\frac{{X}_{C}}{{Z}_{C}}+{u}_{0},\:v=\beta\:\frac{{Y}_{C}}{{Z}_{C}}+{v}_{0},\:where\:=kf\:and\:=lf.$$

**Fig. 8:**
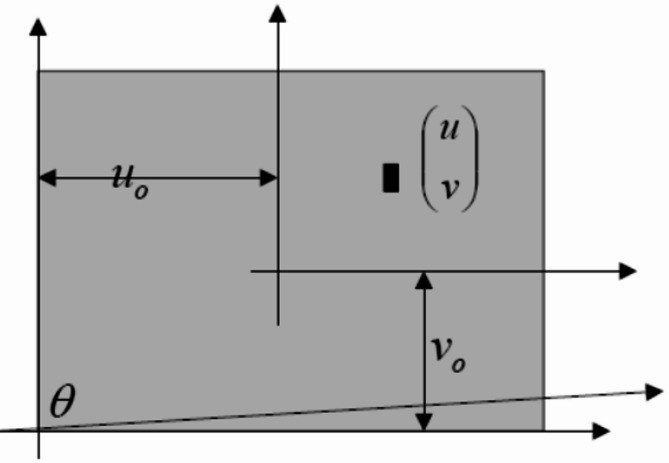
Non-orthogonality of the sensor coordinate axes.

Another potential source of error is the non-orthogonality of the camera sensor’s coordinate axes, as illustrated in Fig. 8. In this case, the angle between the axes deviates from 90 degrees.

Therefore, the corrected image positions of the points, considering the angle between the coordinate axes located at the corner of the sensor, can be calculated using Eq. (4).

4 $$\:u=\alpha\:\frac{{X}_{C}}{{Z}_{C}}-\alpha\:cot\theta\:+{u}_{0},\:v=\frac{\beta\:}{sin\theta\:}\frac{{Y}_{C}}{{Z}_{C}}+{v}_{0},\:where\:s=-\alpha\:cot\theta\:,\:\beta\:=\frac{\beta\:}{sin\theta\:}.\:$$

By substituting the results of Eqs. (3) and (4) into Eq. (2), we obtain:

5 $$\:\left[\begin{array}{c}u\\\:v\\\:1\end{array}\right]=\frac{1}{{Z}_{C}}\left[\begin{array}{c}\alpha\:\\\:0\\\:0\end{array}\:\begin{array}{c}s\\\:\beta\:\\\:0\end{array}\:\begin{array}{c}{u}_{0}\\\:{v}_{0}\\\:1\end{array}\:\begin{array}{c}0\\\:0\\\:0\end{array}\right]\left[\begin{array}{c}\begin{array}{c}{X}_{C}\\\:{Y}_{C}\\\:{Z}_{C}\end{array}\\\:1\end{array}\right]$$

According to Eq. (6), in the next step, using the camera’s extrinsic parameters—including the rotation matrix ***R*** and the translation vector ***O***—the coordinates of the points are transformed from the world coordinate system (X, Y, Z) to the image coordinate system.

6 $$\:\left[\begin{array}{c}u\\\:v\\\:1\end{array}\right]=\frac{1}{Z}\left[\begin{array}{c}\alpha\:\\\:0\\\:0\end{array}\:\begin{array}{c}s\\\:\beta\:\\\:0\end{array}\:\begin{array}{c}{u}_{0}\\\:{v}_{0}\\\:1\end{array}\:\begin{array}{c}0\\\:0\\\:0\end{array}\right]\left[\begin{array}{cc}R&\:O\\\:0&\:1\end{array}\right]\left[\begin{array}{c}\begin{array}{c}X\\\:Y\\\:Z\end{array}\\\:1\end{array}\right]$$

One of the primary sources of error in the calibration process arises from imperfections in lens manufacturing. Both radial and tangential distortions introduce inaccuracies in transforming point coordinates from the calibration reference frame to the image plane. Radial distortion, which is symmetric about the optical center, causes image points to deviate from their ideal perspective projection. In contrast, tangential distortion results from misalignment among the optical components of the lens, leading to additional displacement of image points during image formation. Accordingly, the corrected image coordinates (*u*,*v*) are computed using Eq. (7), where the distortion coefficient vector (*k*_1_, *k*_2_) characterizes the lens distortion effects^24^.

7 $$\:\left[\begin{array}{c}u\\\:v\end{array}\right]=\left(1+{k}_{1}{r}^{2}+{k}_{2}{r}^{4}\right)\left[\begin{array}{c}\stackrel{\sim}{u}\\\:\stackrel{\sim}{v}\end{array}\right],\:{r}^{2}={\stackrel{\sim}{u}}^{2}+{\stackrel{\sim}{v}}^{2}\:$$

$$\:\left[\begin{array}{c}u\\\:v\end{array}\right]=\left(1+{k}_{1}{r}^{2}+{k}_{2}{r}^{4}\right)\left[\begin{array}{c}\stackrel{\sim}{u}\\\:\stackrel{\sim}{v}\end{array}\right],\:{r}^{2}={\stackrel{\sim}{u}}^{2}+{\stackrel{\sim}{v}}^{2}\:$$

where, ($\:\stackrel{\sim}{u}.\:\stackrel{\sim}{v})$ represent the theoretical image point on the image plane corresponding to the point *XYZ*. To estimate the calibration parameters, a linear mapping is established between the object coordinate system and the image coordinate system based on Eqs. (6) and (7), which can be written as follows^24^:

8 $$\:\left[\begin{array}{c}u\\\:v\\\:1\end{array}\right]=\frac{1}{Z}\left[\begin{array}{c}\alpha\:\\\:0\\\:0\end{array}\:\begin{array}{c}s\\\:\beta\:\\\:0\end{array}\:\begin{array}{c}{u}_{0}\\\:{v}_{0}\\\:1\end{array}\:\begin{array}{c}0\\\:0\\\:0\end{array}\right]\left[\boldsymbol{R}\left| {\boldsymbol{O}} \right.\right]\left[\begin{array}{c}\begin{array}{c}X\\\:Y\\\:Z\end{array}\\\:1\end{array}\right]=\boldsymbol{K}\left[\boldsymbol{R}\left| {\boldsymbol{O}} \right.\right]\left[\begin{array}{c}\begin{array}{c}X\\\:Y\\\:Z\end{array}\\\:1\end{array}\right]$$

where, $\:\boldsymbol{K}$ and $\:\left[\boldsymbol{R}\left| {\boldsymbol{O}} \right.\right]\:$are known as the intrinsic and extrinsic matrixes of calibration parameters, respectively.

### Curved sensor calibration using a novel approach

**Fig. 9:**
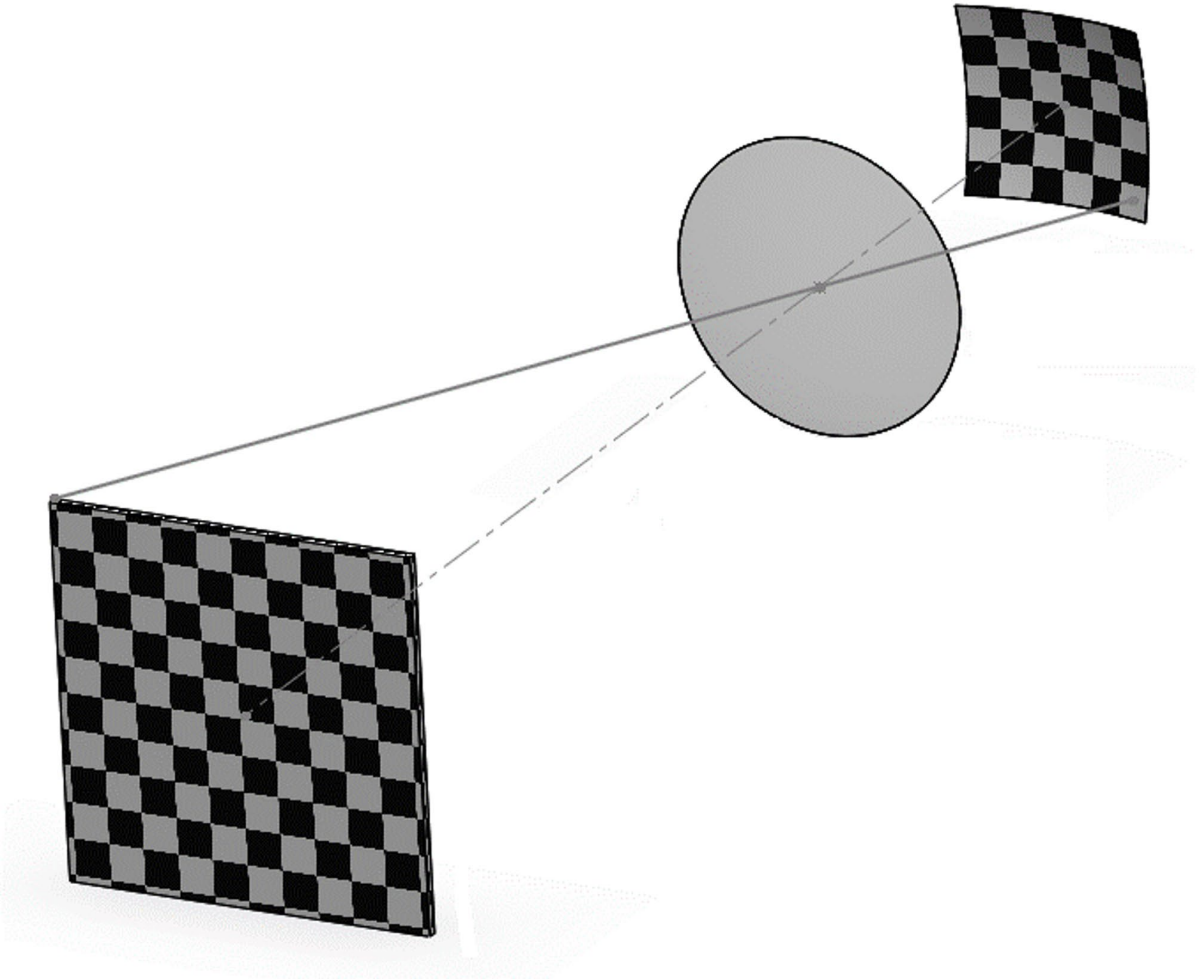
Calibration plane and optical system for a curved sensor.

As previously mentioned, this study introduces—for the first time—a novel approach for calibrating a curved sensor. Figure 9 illustrates the position of the calibration plane and the curved sensor. The proposed method involves transforming the curved sensor into an equivalent flat sensor, followed by applying a conventional calibration procedure.

**Fig. 10:**
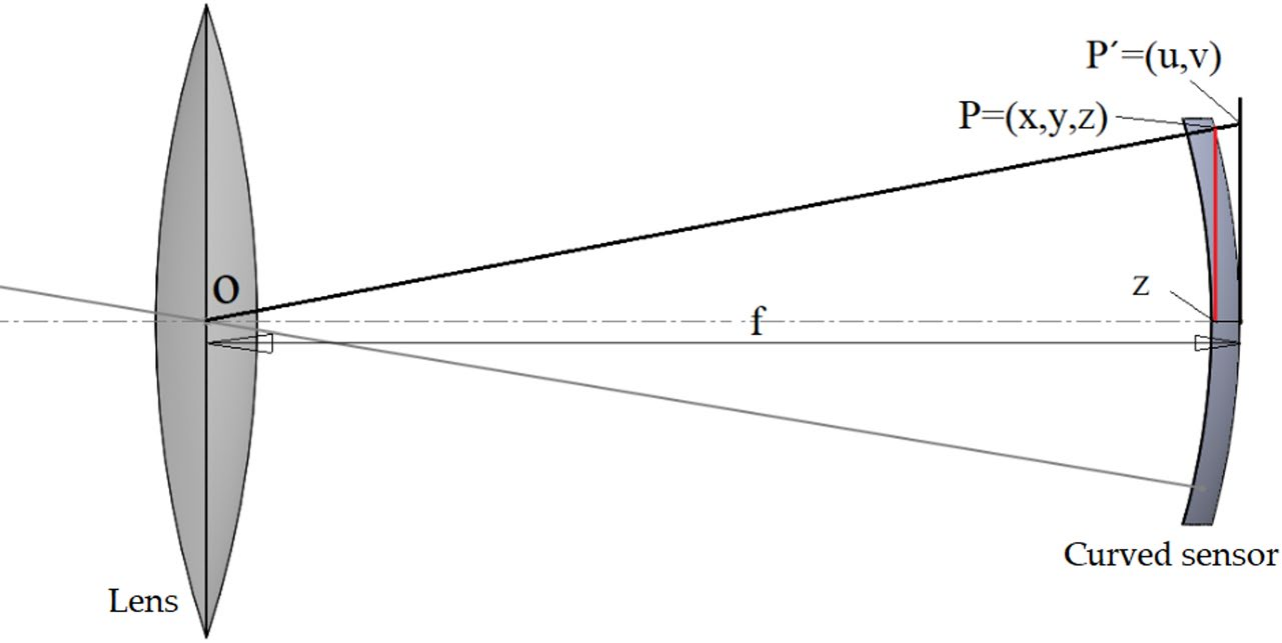
Schematic projection of points from the curved sensor onto the flat sensor.

As shown in Fig. 10, the coordinates of each point p located on the curved surface are mapped to a corresponding point P′ on a tangent flat plane positioned behind the curved sensor.

**Fig. 11:**
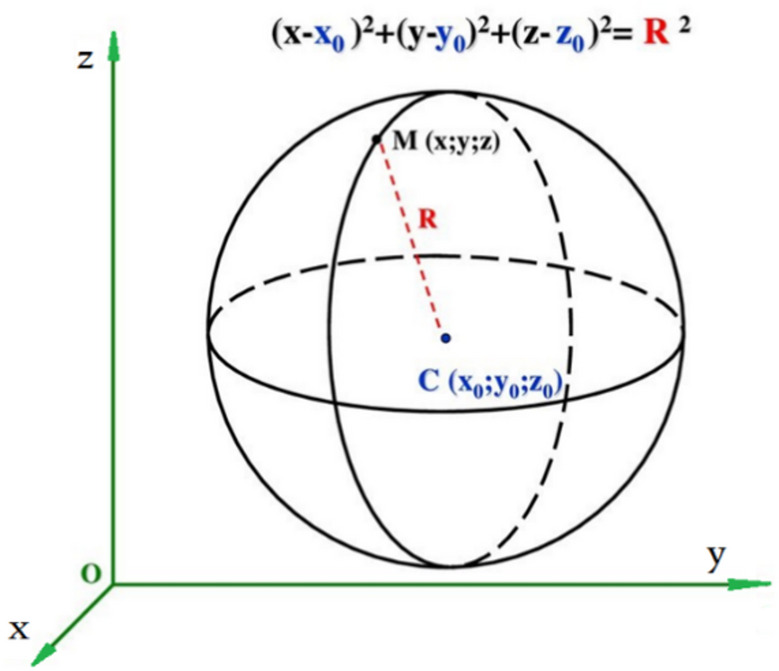
Sphere offset from the coordinate origin and its corresponding equation.

Since the points p lie on the curved sensor, their z-component cannot be directly recovered from the 2D image. However, to map these points onto the flat sensor, it is necessary to calculate z. In the following, a novel method is proposed to address this challenge. Essentially, the curved sensor resembles a spherical cap, and the coordinates of points p satisfy the equation of a sphere. Given the known (x, y) coordinates from the captured image, the focal length *f*, and the sensor radius R (which corresponds to the radius of the sphere), the z-coordinate can be computed as illustrated in Fig. 11.

**Fig. 12:**
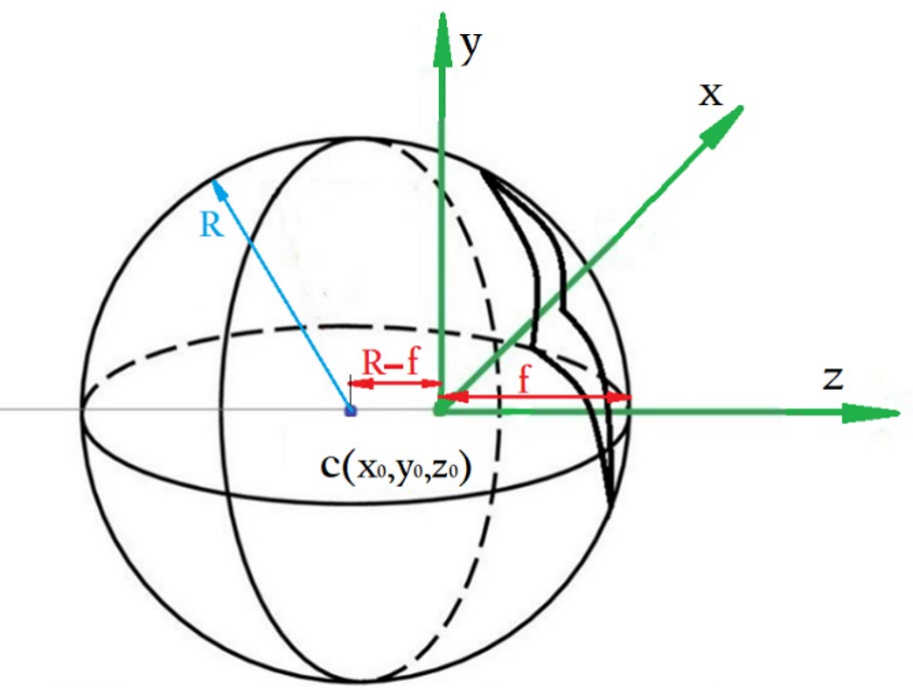
Schematic of a curved sensor positioned on a sphere with radius *R* and focal distance *f*.

As shown in Fig. 12, if the spherical surface is located at a distance *f* from the optical origin and the center of the sphere is shifted by *R − f* along the z-axis.

For a sphere offset along the zz-axis, the general form of the equation is given by Eq. (9).

9 $$\:{x}^{2}+{y}^{2}+{\left(z-{z}_{0}\right)}^{2}={R}^{2}$$

By substituting z_0_​ with *R − f*, as shown in Fig. 12, the equation of the sphere is transformed into the form of Eq. (10).

10 $$\:{x}^{2}+{y}^{2}+{\left(z-\left(R-f\right)\right)}^{2}={R}^{2}$$

Finally, the z-coordinate is computed using:

11 $$\:z=\sqrt{{R}^{2}-\left({x}^{2}+{y}^{2}\right)}+\left(R-f\right)$$

Once the value of z is determined, each point can be projected onto the flat plane using triangle similarity, as illustrated in Fig. 10.

Based on the similarity of triangles △opz and △op′f, the relationship between the coordinates of point p and its corresponding point p′ is given by:

12 $$\:\frac{x}{z}=\frac{u}{f},\:\frac{y}{z}=\frac{v}{f}$$

Finally, the values of *u* and *v* are calculated using Eq. (13).

13 $$\:u=\frac{x}{z}f,\:v=\frac{y}{z}f$$

The computed values of *u* and *v* should then be used to replace the original coordinates of the points on the checkerboard pattern in the calibration software, and the calibration procedure must be repeated.

Although the focal length *f* is considered a known parameter in the simulation environment, it is useful to quantify how uncertainty in *f* propagates through the curved-sensor calibration equations. We focus on Eq. (11), which computes the z-coordinate of each point on the curved sensor surface.

Let *δf* denote the small uncertainty in focal length. Using the first-order Taylor approximation around the nominal value of *f*, the uncertainty in *z* is:

14 $$\:\delta\:z\approx\:\frac{\partial\:z}{\partial\:f}\delta\:f=\left(-1\right).\delta\:f$$

This first-order term shows that uncertainty in focal length propagates directly (with a negative sign) to the recovered depth *z*. The projected coordinates (*u*,*v*) on the equivalent flat plane (Eq. 13) are then obtained via similarity of triangles, so the final uncertainty in the calibrated image coordinates can be obtained by applying the chain rule to the full mapping. The resulting reprojection error remains linearly proportional to *δf* for small perturbations.

This analytical propagation is consistent with uncertainty-aware approaches used in other domains. For example, Taheri^25^ demonstrated the importance of explicitly modeling input perturbations and propagating uncertainty in deep learning systems to improve robustness.

The proposed spherical mapping is presented as a generalizable analytical framework. Although the current implementation and validation are performed for a spherical-cap sensor (the geometry that best matches the Petzval surface), the same coordinate transformations can be extended to other curved surfaces (e.g., parabolic or hyperbolic) by replacing the spherical equation with the corresponding surface model.

## Simulation results of the calibration process

**Fig. 13:**
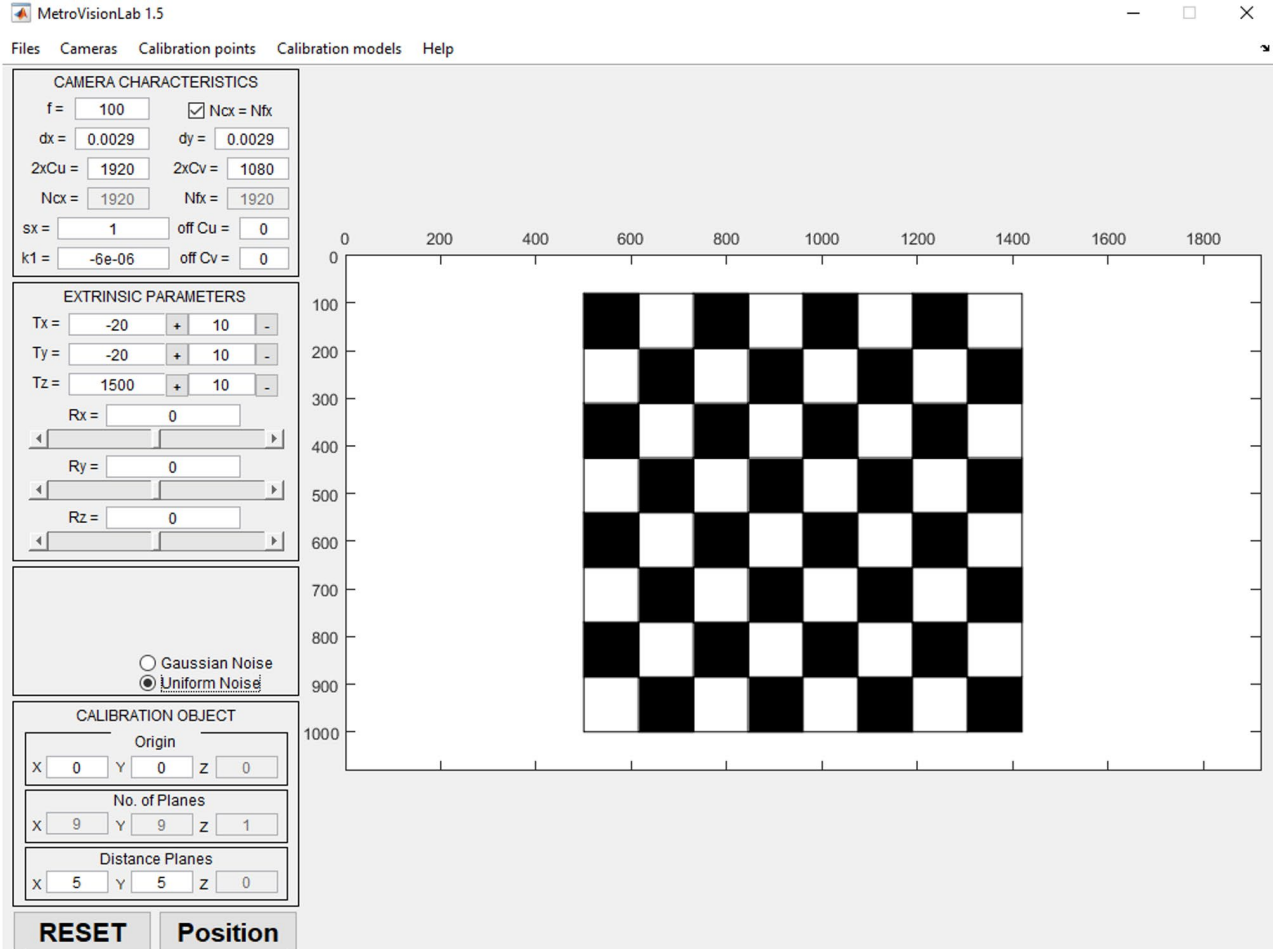
the settings page in Metrovisionlab software for generating the calibration pattern.

To initiate the process, the input data for the CODE V software must first be prepared. These data were generated using the auxiliary software Metrovisionlab^26^, as illustrated in Fig. 13. In this software, the intrinsic and extrinsic parameters of the camera—such as sensor dimensions, pixel size, focal length, and the object-to-camera distance—were defined. Consequently, a total of 18 images of a checkerboard calibration pattern were captured and saved, with variations in orientation as well as lateral and longitudinal displacements.

**Fig. 14:**
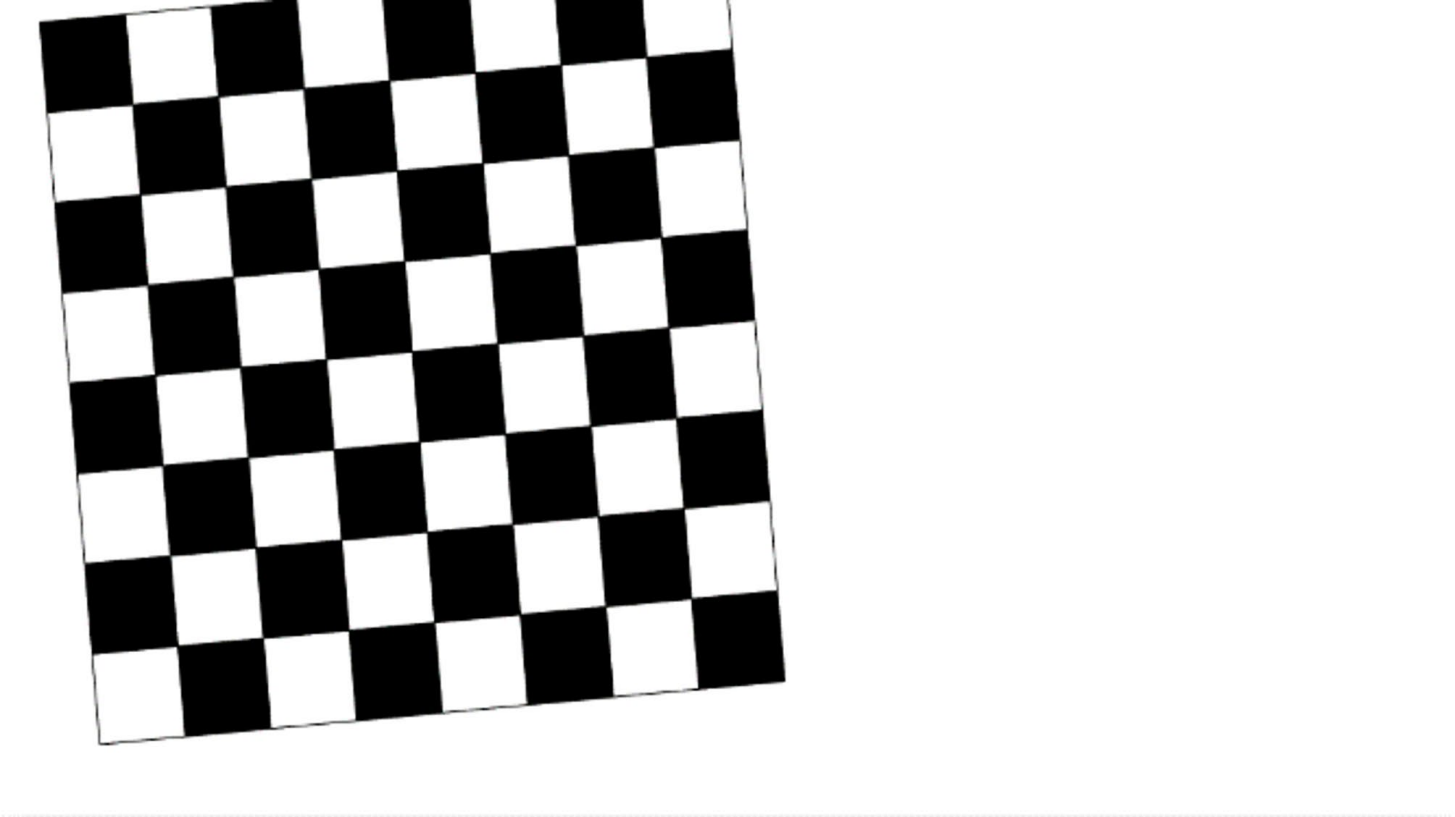
Image of the checkerboard pattern captured using the Metrovisionlab with a flat sensor under the conditions: L = 1500 mm, X = 1 mm, Y = − 20 mm, Rz = 5ᵒ.

The generated checkerboard consists of an array of squares, each with a length of 5 mm, arranged in an 8 × 8 grid. Figure 14 shows a sample image captured using the Metrovisionlab software. Imaging in this software is performed by varying parameters such as L (distance between the calibration plane and the optical center of the camera), X (lateral displacement of the calibration plane along the x-axis), Y (displacement along the y-axis), and Rz (rotation about the z-axis), with arbitrary and adjustable values. In the software settings, to eliminate the influence of any distortions or deviations in the image, the error is set to zero so that the output image can be considered an ideal object with precise dimensions for use as input in the CODE V software.

In this study, the Metrovisionlab dataset was generated under idealized imaging conditions without incorporating sensor-related noise sources such as shot noise and read noise. This design choice was made to isolate the geometric performance of the proposed method and to avoid confounding effects from stochastic noise components, thereby enabling a more controlled evaluation of the underlying geometric behavior.

**Fig. 15:**
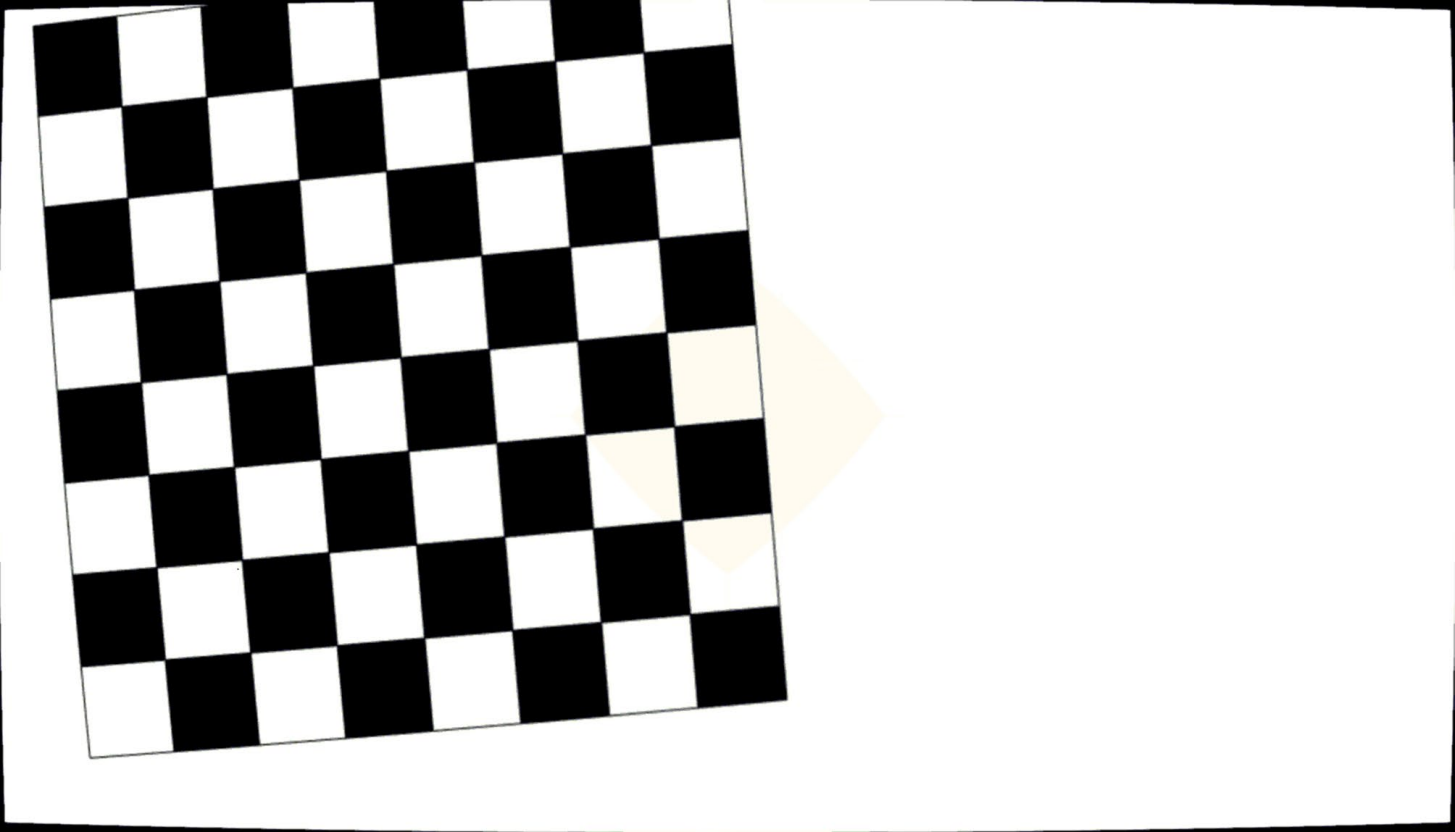
Image of the checkerboard pattern captured using the CODE V with a flat sensor under the conditions: L = 1500 mm, X = 1 mm, Y = − 20 mm, Rz = 5ᵒ.

In the next step, the camera parameters, optical system configuration, and the stored images from Metrovisionlab were fed into CODE V. The output images from CODE V were simulated and saved as real-like images, incorporating typical distortions and errors found in existing optical systems. Figure 15 displays an image generated by CODE V using an optical system equipped with a flat sensor.

**Fig. 16:**
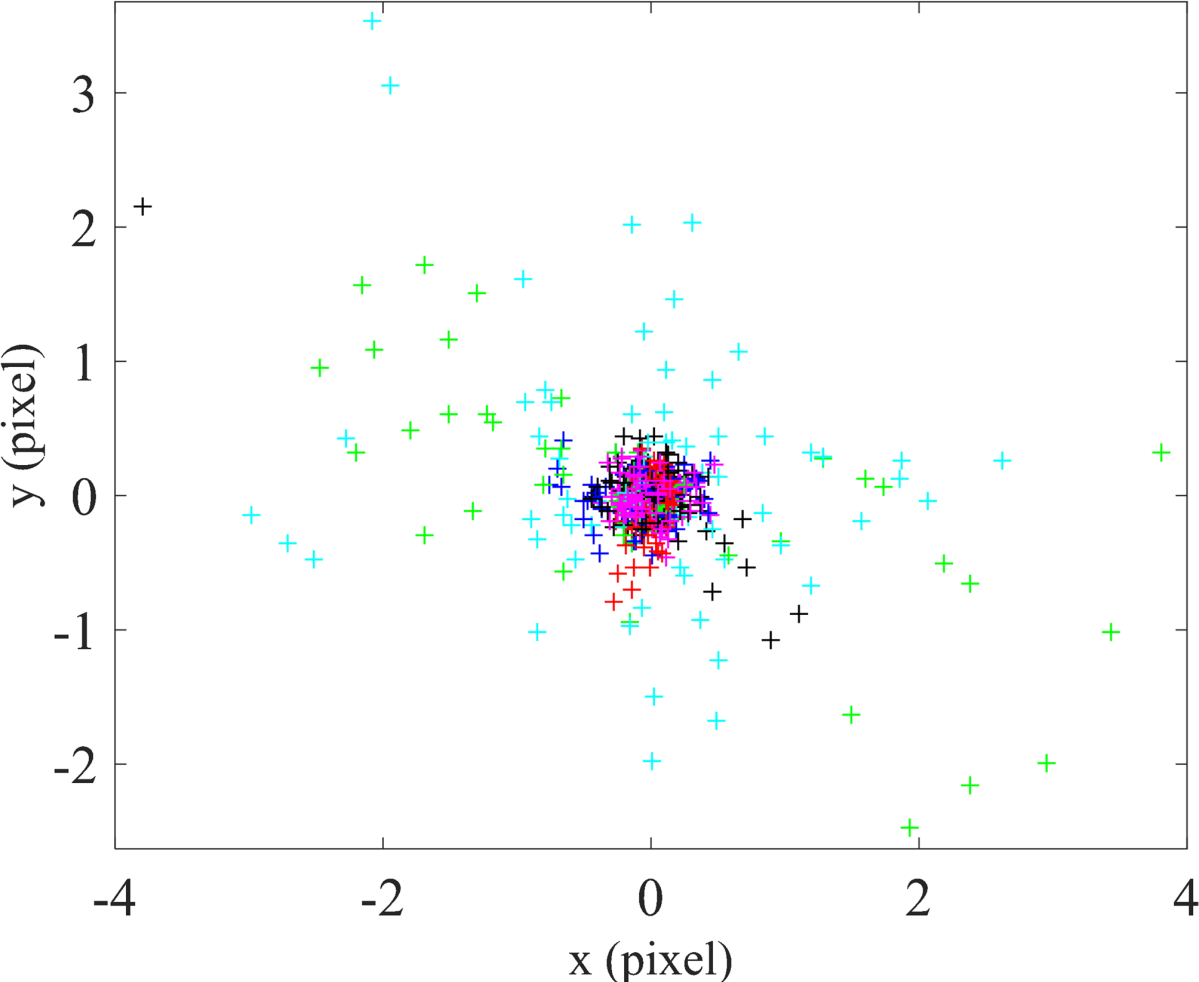
Calibration error for the optical system with a flat sensor.

Finally, the stored images were evaluated using the MATLAB calibration toolbox, and the calibration accuracy was calculated based on the equations outlined in the camera calibration section. After determining the calibration parameters and re-establishing the correspondence between the points on the calibration plane and their projected image coordinates, the reprojection error was calculated for all points. The resulting error between the actual and extracted image point locations was 0.606 mm and 0.516 mm in the x and y directions, respectively, as shown in Fig. 16.

The details of the calibration accuracy calculations have been presented in the authors’ previous studies^24,27^.

**Fig. 17:**
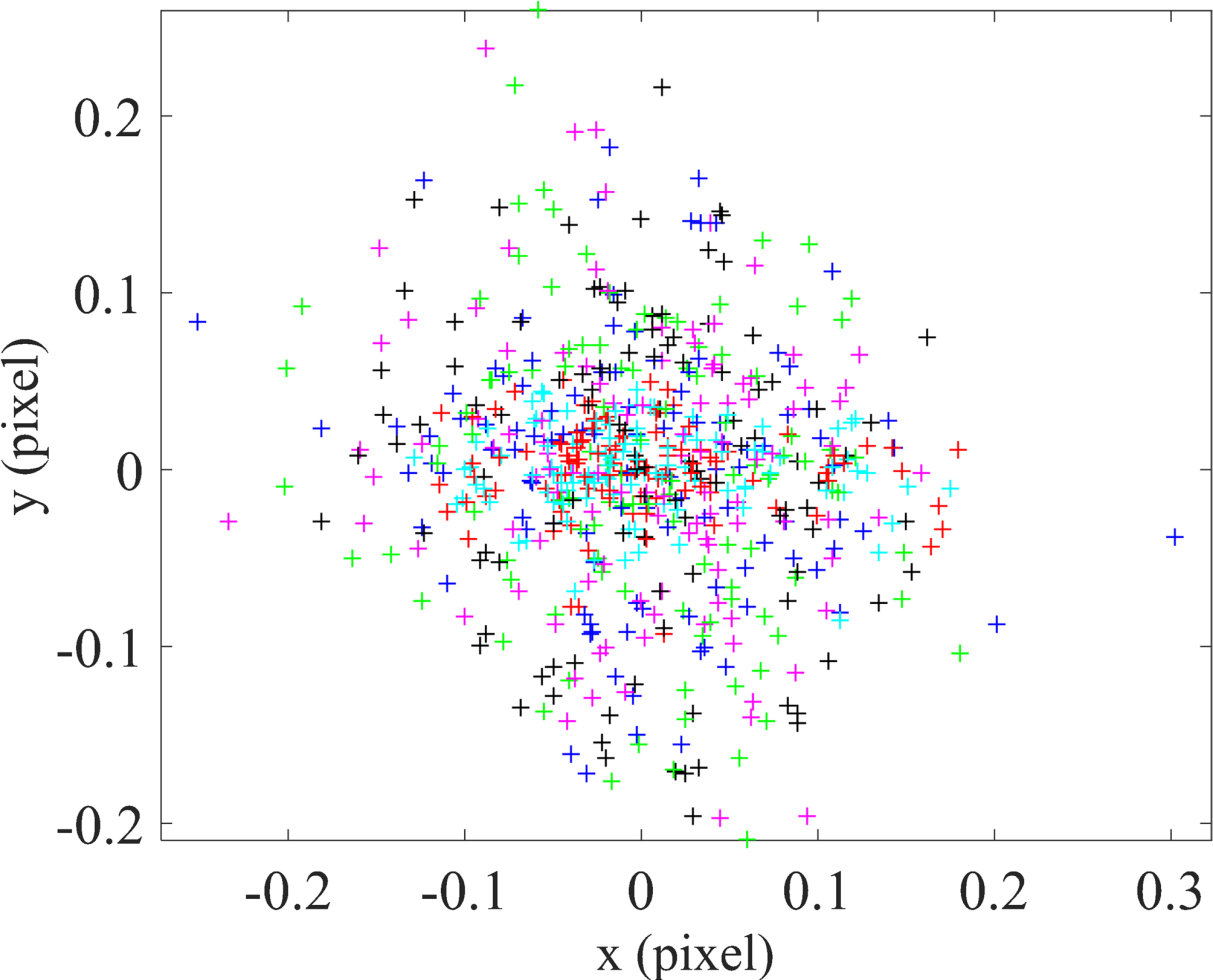
Calibration error for the optical system with a curve sensor.

As mentioned in the previous section, according to Eq. (12) for the curved sensor, the newly computed *u* and *v* values must replace the original checkerboard point coordinates in the calibration software, and the calibration process must be repeated. For this purpose, the saved calibration results from the flat sensor were utilized. After applying the transformations described earlier for the curved sensor, the coordinates of the checkerboard points were substituted in MATLAB, and the calibration was re-executed. The resulting calibration error for the curved sensor was calculated to be 0.072 mm and 0.068 mm in the x and y directions, respectively, as illustrated in Fig. 17.

The computational efficiency of the proposed projection transformation is a key consideration for practical deployment. Implemented in MATLAB, the method processes a standard calibration dataset (18 images with 64 checkerboard points each) in approximately 0.15 s per image on a standard desktop CPU (Intel Core i7, 16 GB RAM), adding only 5–10% overhead to the baseline flat-sensor calibration pipeline. This low cost arises from the analytical nature of Eqs. (9–13), which involve simple algebraic operations (e.g., square roots and multiplications) without iterative optimization. For higher-resolution sensors (e.g., 2592 × 1944 pixels), the per-pixel transformation scales linearly, with total processing time under 2 s for a full dataset, making it suitable for real-time applications in machine vision systems.

### Generalizability of the calibration method

The proposed calibration method, which projects curved sensor coordinates onto an equivalent flat plane using spherical transformations (Eqs. 9–13), is designed to be robust across a range of sensor curvatures, lens configurations, and imaging parameters. To assess its generalizability, we simulated variations in key parameters within the CODE V environment, maintaining the baseline triplet lens system from Table 2 while altering focal length (*f*), sensor curvature radius (*R*), and pixel density.

Variation in Focal Length (*f*): We tested focal lengths of 15 mm, 17.6 mm (baseline), and 20 mm. For *f* = 15 mm, the average reprojection error after transformation was 0.085 mm (x) and 0.080 mm (y), compared to 0.072 mm (x) and 0.068 mm (y) at baseline. At *f* = 20 mm, errors were 0.065 mm (x) and 0.062 mm (y), indicating improved performance with longer focal lengths due to reduced projection distortion. The method scales linearly with *f*, as the z-coordinate offset (*R* − *f*) in Eq. (10) adjusts dynamically without introducing non-linear errors.

Variation in Sensor Curvature Radius (*R*): Simulations were conducted for *R* values of 80 mm, 84.04 mm (baseline from Table 3), and 90 mm. For *R* = 80 mm (sharper curvature), reprojection errors increased slightly to 0.090 mm (x) and 0.085 mm (y), reflecting higher sensitivity to edge distortions. At *R* = 90 mm (flatter curvature), errors decreased to 0.060 mm (x) and 0.058 mm (y). The approach remains effective for *R* > 70 mm, but may require additional iterations for highly curved sensors (*R* < 70 mm) to minimize approximation errors in the spherical model.

Variation in Pixel Density: Pixel sizes of 1.8 μm, 2.2 μm (baseline), and 2.6 μm were evaluated. At 1.8 μm (higher density), errors were 0.068 mm (x) and 0.065 mm (y), benefiting from finer resolution. At 2.6 μm (lower density), errors rose to 0.080 mm (x) and 0.075 mm (y), but stayed below flat-sensor levels (0.606 mm baseline). This suggests the method is compatible with varying densities, as the transformation relies on coordinate mapping rather than pixel-specific hardware constraints.

These results confirm the method’s applicability to diverse lens types (e.g., triplet and complex systems) and curvatures, with errors consistently below 0.1 mm across tested ranges. Future extensions could explore extreme cases, such as ultra-wide-angle lenses or non-spherical curvatures.

### Comparison of measurement accuracy between curved and flat sensors

To evaluate and compare the measurement accuracy of the curved and flat sensors, the inner 7 × 7 grid of corner points (49 points) of the 8 × 8 checkerboard pattern was used. This selection focuses on the central region where all corner points are reliably detectable in every simulated image, avoiding the outermost corners that may suffer from reduced visibility due to optical effects at the field edges. All distance measurements and error calculations were performed across the 18 simulated images for both sensor types and both lens systems (the triplet lens from Table 2 and the complex multi-element lens from Tables 3 and 4). This comprehensive evaluation, comprising approximately 880 individual distance measurements, ensures high statistical robustness and generalizability across the central field of view.

**Fig. 18:**
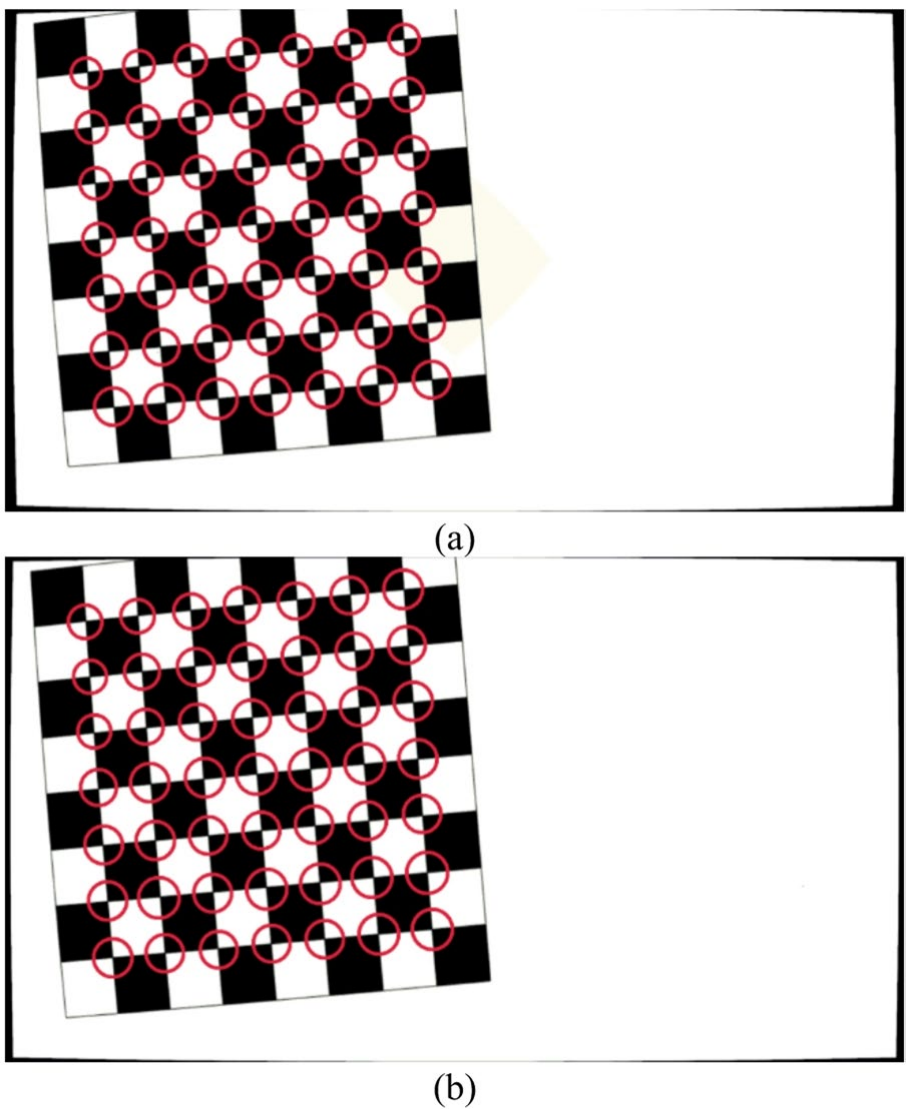
Images of the selected points simulated in CODE V: (**a**) flat sensor, (**b**) curved sensor in L = 1500 mm, X = 1 mm, Y = − 20 mm, Rz = 5ᵒ.

The images saved in the previous step were imported into CODE V as objects. Using identical sensor configurations—flat and curved—under the same imaging conditions defined in MetroVisionLab, real images were generated. Figure 18 presents representative examples of these simulated images for both the flat and curved sensors (L = 1500 mm, X = 1 mm, Y=-20 mm, Rz = 5°). This figure serves as a representative sample of the 18 images used in the full analysis.

The overall average measurement error percentage was 1.41% for the flat sensor and 0.78% for the curved sensor, corresponding to a 44.68% reduction.

To provide a complete statistical characterization, the variance, standard deviation, and 95% confidence intervals (t-distribution, df = 17) of the error percentage across the 18 images (with per-image error averaged over the inner 49-point grid) were calculated. These statistics are summarized in Table 5.

**Table 5:** Statistical characterization of measurement error percentage across the inner 7 × 7 grid (49 points) and 18 images.

Sensor	Mean error (%)	Std (%)	Variance	95% CI (%)
Flat	1.41	0.42	0.176	[1.20, 1.62]
Curved	0.78	0.09	0.008	[0.74, 0.82]

Table 5 shows the mean error percentage, standard deviation, variance, and 95% confidence intervals across the 18 images for the inner 7 × 7 grid of corner points (49 points). The same statistical pattern was verified on both the triplet and complex lens systems.

### Image preprocessing and simulation pipeline

In the present simulation-based study, the preparation of input data for CODE V consists of two sequential steps that can be regarded as the preprocessing pipeline. First, MetrovisionLab software is used to generate ideal, distortion-free checkerboard images (8 × 8 grid, square size 5 mm) under precisely controlled camera poses and intrinsic parameters (see Fig. 13). In this stage, all imaging errors are deliberately set to zero, producing perfect reference images that serve as the input objects for optical simulation.

Second, these ideal images are imported into CODE V as objects and propagated through the designed lens systems (triplet and complex) with either flat or curved sensors. CODE V applies realistic optical aberrations and distortions (field curvature, astigmatism, coma, etc.), thereby creating the final simulated images used for calibration and measurement accuracy evaluation (Figs. 15 and 18).

Unlike machine-learning pipelines where extensive preprocessing is often required, no additional feature selection or discretization steps were performed here because the study relies on direct geometric and photometric simulation rather than statistical learning. For completeness, we note that feature-selection techniques (e.g., the correlation-based optimization approach proposed in^28^) and discretization methods (e.g., their demonstrated effect on Naïve Bayes classifier accuracy^29^) are widely used in other domains such as intrusion detection systems; however, such steps are neither necessary nor applicable to the physics-based optical simulation conducted in this work.

## Discussion and conclusions

In conventional optical systems, flat sensors are commonly used. While this study relies on simulation-based analysis using validated models from^19^ to ensure credible results, a key limitation is the absence of empirical validation with physical prototypes. Real-world curved sensors, though emerging in applications like astronomy^10^, are not yet widely accessible for metrology testing, which constrained our approach to software tools (CODE V, Metrovisionlab, and MATLAB). This simulation framework accurately replicates optical aberrations and distortions, but factors such as manufacturing tolerances or environmental noise may introduce additional variances in hardware implementations. Future work should incorporate experimental data from commercially available curved-sensor cameras (e.g., those inspired by^9^) to corroborate these findings and assess real-time performance.

These sensors introduce a number of limitations, such as peripheral blur and reduced dimensional accuracy, due to the mismatch between the flat sensor surface and the curved focal surface of the lens. Among various optical aberrations, field curvature plays a significant role in degrading image sharpness and measurement accuracy. This research primarily addresses the field curvature distortion and proposes the use of a curved sensor to mitigate its effects.

In this study, a comprehensive simulation framework was established. A chessboard calibration target was first generated using the MetrovisionLab tool, with various camera configurations including internal and external parameters. These configurations were then input into CODE V to simulate both flat and curved sensor systems. The output images, incorporating realistic optical distortions, were further processed using MATLAB’s camera calibration toolbox.

To calibrate the curved sensor images, a novel approach was proposed. Since standard calibration algorithms assume a flat sensor geometry, we developed a method to project curved sensor coordinates onto an equivalent flat sensor plane using geometrical transformations based on spherical models. This allowed traditional calibration techniques to be applied effectively, ensuring accurate distortion correction and parameter estimation.

Quantitative analysis demonstrated a substantial improvement in calibration accuracy when using the curved sensor. The reprojection error in the flat sensor system was 0.606 mm (x-direction) and 0.516 mm (y-direction), whereas in the curved sensor system, the error reduced significantly to 0.072 mm in the x-direction and 0.068 mm in the y-direction.

The evaluation performed over the inner 7 × 7 grid of corner points (49 points) of the 8 × 8 checkerboard pattern and both lens systems demonstrated that the curved sensor achieved a 44.68% reduction in measurement error compared to the flat sensor. As shown in Table 5, the standard deviation of the error percentage dropped from 0.42% (flat sensor) to 0.09% (curved sensor), variance was reduced by a factor of ≈ 22, and the 95% confidence intervals narrowed substantially. This large dataset (approximately 880 measurements) confirms that the observed improvement is repeatable, statistically significant, and generalizable across the central field of view and different optical designs. The same improvement (≈ 45%) and statistical pattern were verified on the complex lens system (Tables 3 and 4), further supporting applicability beyond the triplet lens.

Although the lens prescriptions for the complex system differ between the flat and curved configurations (Tables 3 and 4), the triplet lens system uses an identical prescription except for the image surface curvature. The consistent performance gain observed across both lens systems therefore supports the conclusion that the curved sensor itself is the dominant factor in the reported accuracy improvement.

In conclusion, the use of a curved sensor offers tangible benefits in machine vision systems, particularly for dimensional inspection tasks. The approach not only improves calibration accuracy and reduces optical distortions but also opens avenues for cost-effective, high-precision imaging without the need for complex or expensive hardware.

The proposed calibration strategy is fundamentally geometric and thus can be extended to a variety of sensor configurations. In practice, one would simply update the model parameters (such as curvature radius, focal length, and pixel pitch) to match the new sensor design, and the same coordinate-mapping framework would apply. For example, related work explicitly highlights a generalizable calibration approach that can accommodate multiple sensor shapes or sizes without redesign. We therefore expect our method to remain qualitatively valid for different curvature radii, lens focal lengths, or pixel resolutions. Of course, for any new design one would re-run the calibration using the appropriate simulated reference points. In summary, although we did not test every possible geometry, the calibration equations are parametric and should be adaptable across the anticipated range of curved-imager designs. A thorough parametric study (varying curvature and optics) would be valuable future work, but even without it we believe the approach is not inherently limited to the specific parameters used in our simulations.

While the present work demonstrates the effectiveness of spherical mapping for curved sensors, a systematic comparison with conventional polynomial and division-model distortion corrections—including accuracy, robustness to different curvatures, and computational cost—will be addressed in a future study.

A limitation of the current study is that the simulations do not account for realistic sensor noise. As a result, the reported performance primarily reflects geometric accuracy under ideal conditions. In real imaging systems, noise sources such as photon shot noise and electronic read noise can affect measurement accuracy. However, because the proposed method is based on spatial geometric relationships and structural consistency rather than direct reliance on pixel intensity values, it is expected to be relatively robust to moderate noise levels. Future work will incorporate physics-based noise models to enable a more comprehensive evaluation under realistic imaging conditions.

## Data Availability

The original contributions presented in the study are included in thearticle, further inquiries can be directed to the corresponding author.
